# Bleeding disorders and postpartum hemorrhage by mode of delivery: a retrospective cohort study

**DOI:** 10.1016/j.rpth.2023.100166

**Published:** 2023-04-26

**Authors:** Bonnie Niu, Lisa Duffett, Darine El-Chaâr, Alan Tinmouth, Tzu-Fei Wang, Roy Khalife

**Affiliations:** 1 Faculty of Medicine, University of Ottawa, Ottawa, Ontario, Canada; 2 Department of Medicine, Division of Hematology, Ottawa Hospital Research Institute, University of Ottawa, Ottawa, Ontario, Canada; 3 Department of Obstetrics and Gynecology, Ottawa Hospital Research Institute, University of Ottawa, Ottawa, Ontario, Canada

**Keywords:** blood coagulation disorders, hemophilia A, postpartum hemorrhage, pregnancy, von Willebrand disease

## Abstract

**Background:**

Pregnant persons with bleeding disorders and their potentially affected newborns are at a higher risk of peripartum bleeding complications. The safest mode of delivery for persons with bleeding disorders remains debated, leading to uncertainties in decision-making between the patient and her multidisciplinary team.

**Objectives:**

This study aimed to describe maternal outcomes for pregnant persons with bleeding disorders by mode of delivery and to examine whether postpartum hemorrhage (PPH) and neonatal hemorrhagic manifestations are associated with the mode of delivery.

**Methods:**

We collected retrospective data on pregnant persons with bleeding disorders who delivered at a single center from 2010 to 2021. Descriptive statistics, Fisher exact test, and odds ratios were used for analysis.

**Results:**

A total of 82 pregnancies in 56 subjects were included. Hemophilia A and von Willebrand disease represented the largest cohort, at 30% (17/56) each. Overall rates of primary and secondary PPH were 7.3% (6/82) and 17.4% (12/69), respectively. We did not find a statistically significant difference between mode of delivery and PPH. Upon comparing vaginal and cesarian deliveries, we found an odds ratio of 0.7 (95% CI, 0.1-3.4) for primary PPH and 2.6 (95% CI, 0.4-16.4) for secondary PPH. One male newborn with severe hemophilia A was treated for a suspected intracranial hemorrhage.

**Conclusion:**

In our cohort, high rates of PPH remained an important complication for pregnant persons with bleeding disorders. There was no significant difference in PPH based on modes of delivery. The small sample size likely limited the power of our study, and consequently, future larger studies are needed.

## Essentials

• Peripartum management of persons with bleeding disorders requires additional research efforts.
• We described maternal and neonatal outcomes from a single Canadian tertiary care center.
• Persons with bleeding disorders had high rates of secondary postpartum hemorrhage.
• We found no significant difference between rates of postpartum hemorrhage and mode of delivery.

## 1 Introduction

Pregnant persons with bleeding disorders and their potentially affected newborns are at higher risk of peripartum bleeding complications compared to the general population [1,2]. The safest mode of delivery for pregnant persons with bleeding disorders remains debated and primarily driven by obstetrical indications [3,4]. While planned cesarian delivery (CD) in persons without bleeding disorders may offer a lower overall incidence of postpartum hemorrhage (PPH) [5] and less short-term adverse outcomes than vaginal deliveries, the risk of bleeding increases with repeated CDs due to higher risk of placental abnormalities and surgical complexities [6]. Spontaneous vaginal delivery (SVD) offers the least invasive approach and decreases the need for CD in subsequent deliveries [3,[7], [8], [9]], but it may complicate laboratory monitoring and factor concentrate administration essential for safe deliveries in persons with bleeding disorders. Induction of labor (IOL) involves using planned techniques to stimulate uterine contractions and achieve delivery, which may include cervical ripening, oxytocin, or amniotomy. Compared to planned CD, IOL can provide similar predictability while minimizing the risks of bleeding and associated morbidity [[7], [8], [9]]. However, IOL was shown to increase the risk of peripartum bleeding events in pregnant persons on anticoagulation [10].

Managing pregnant persons with bleeding disorders is challenging due to insufficient evidence to guide complex decision-making. There are debates among experts on whether to choose CD or vaginal delivery without instrumentation and prolonged labor [3,4]. This results in clinical uncertainties and variable practices that affect shared decision-making, preparedness, and clinical outcomes. Randomized controlled trials are unlikely due to the rarity of bleeding disorders [11]; thus, observational studies are crucial to inform practice and areas for further research. This study aimed to describe the association between mode of delivery and maternal and neonatal outcomes, specifically PPH and neonatal intracranial hemorrhage, in pregnant persons with bleeding disorders.

## 2 Methods

We conducted a retrospective cohort study of pregnant persons with bleeding disorders managed at the Ottawa Regional Bleeding Disorders program at The Ottawa Hospital (TOH). The Ottawa Health Science Network Research Ethics Board approved all study procedures (ID: 20210313-01H).

The study included subjects who met 2 criteria: 1) a known bleeding disorder diagnosis, including hemophilia A or B, von Willebrand disease (VWD), rare bleeding disorders, or inherited platelet function defects, and 2) delivered at TOH between January 1, 2010, and July 15, 2021 (further details in Supplementary Material 1). Persons with bleeding disorders not otherwise specified or with bleeding of an unknown cause were excluded.

Data were collected on maternal age and gestational age at delivery, type of bleeding disorder and its characteristics, mode of delivery, and peripartum hemostatic management. Each delivery was recorded as a separate event. Maternal outcomes were recorded, including primary and secondary PPH, as defined by the American College of Obstetrics and Gynecology [12]. Primary PPH was blood loss ≥1000 mL with signs and symptoms of hypovolemia within 24 hours of delivery regardless of the route of delivery or blood loss >500 mL in a vaginal delivery. Secondary PPH was any abnormal or excessive vaginal bleeding beyond 24 hours and up to 12 weeks postpartum, as documented in clinical notes. Other recorded outcomes included maternal death, morbidity, length of hospital stay, and number of packed red blood cell (PRBC) or platelet transfusions, when applicable. Neonatal outcomes were also collected, including neonatal death, morbidity, length of hospital stay, and transfer to a pediatric hospital or the neonatal intensive care unit.

Descriptive statistics were used to summarize the baseline characteristics of the cohort. Missing values were not omitted from the data analysis. Fisher exact test was used to examine the significance of the association between the mode of delivery and PPH. Odds ratios (ORs) were used to analyze the associations between PPH and vaginal or cesarian deliveries and between PPH and planned or unplanned deliveries.

## 3 Results and Discussion

Eighty-two deliveries in 56 persons with bleeding disorders had care at TOH during the study period. The mean (±SD) maternal age was 31.4 (±4.5) years. The mean (±SD) gestational age was 38.6 (±3.0) weeks. Hemophilia A and VWD type 1 were the most common diagnoses, with each representing 30.4% (17/56) of the cohort (Table 1). All subjects had pre-established diagnoses, except 3 who were diagnosed following their first pregnancy (2 VWD type 1 and 1 factor [F]XIII deficiency). SVD was the most common mode of delivery (43.9%), followed by IOL with intended vaginal delivery (29.3%), planned CD (17.0%), and emergent CD (9.8%) (Table 1). In the latter group, 2 subjects failed IOL and required emergent CD.

**Table 1:** Baseline characteristics of the patients.

Characteristics	No. (%)
Maternal age at time of delivery (y)	*N* = 82 deliveries
<20	1 (1.2)
20-24	3 (3.7)
25-29	24 (29.3)
30-34	35 (42.7)
>35	19 (23.2)
Gestational age at time of delivery (wk)	*N* = 82 deliveries
32 + 0-36 + 6	5 (6.1)
37 + 0-39 + 9	50 (61.0)
≥40	27 (32.9)
Mode of delivery	*N* = 82 deliveries
Spontaneous vaginal delivery	36 (43.9)
Induction of labor with intended vaginal delivery	24 (29.3)
Planned cesarian delivery	14 (17.0)
Emergent cesarian delivery	8 (9.8)
Bleeding disorders	*N* = 56 patients
Hemophilia	23 (41.1)
Hemophilia A	17 (30.4)
Hemophilia B	6 (10.7)
VWD	23 (41.1)
VWD type 1	17 (30.4)
VWD type 2 (non-B subtypes)	4 (7.1)
VWD type 2B	2 (3.6)
Combined hemophilia A + VWD	3 (5.4)
FVII deficiency	3 (5.4)
FXI deficiency	1 (1.8)
FXIII deficiency	2 (3.6)
Platelet type: VWD	1 (1.8)

VWD, von Willebrand disease.

Table 2 shows primary and secondary PPH frequency by bleeding disorder type. Overall rates were 7.3% (6/82) for primary PPH and 17.4% (12/69) for secondary PPH. Primary PPH rates were 2.8% (1/36) for SVD, 12.5% (3/24) for IOL, 12.5% (1/8) for emergent CD, and 7.1% (1/14) for planned CD (Supplementary Material 2). Two obstetrical causes were identified for primary PPH. Data for secondary PPH were unavailable for 13 deliveries due to a lack of documentation (Supplementary Material 3). Most secondary PPH occurred between 24 hours and 6 weeks after delivery, except for 1 subject with bleeding persisting to the seventh week. Secondary PPH rates were 18.8% (6/32) for SVD, 22.7% (5/22) for IOL, 14.3% (1/7) for emergent CD, and none (0/8) in the planned CD group (Supplementary Material 2). No statistically significant difference was found between modes of delivery and PPH, either primary or secondary. Upon comparing vaginal deliveries to CDs, the OR was 0.7 (95% CI, 0.1-3.4) for primary PPH and 2.6 (95% CI, 0.4-16.4) for secondary PPH (Table 3). Upon comparing unplanned (SVD and emergent CD) to planned (IOL and planned CD) deliveries, the OR was 0.5 (95% CI, 0.1-2.3) for primary PPH and 1.1 (95% CI, 0.3-3.7) for secondary PPH (Table 3). Table 4 describes each PPH event, including the type of bleeding disorder, coagulation profile, mode of delivery, and peripartum hemostatic therapies.

**Table 2:** Frequency of primary and secondary PPH by type of bleeding disorders.

Bleeding disorders	Total no. of women *N* = 56	No. of deliveries with data available for primary PPH *N* = 82	Frequency of primary PPH *n*/*N* (%)	No. of deliveries with data available for secondary PPH *N* = 69	Frequency of secondary PPH *n*/*N* (%)
Hemophilia	23	32	1/32 (3.1%)	24	4/24 (16.7%)
Hemophilia A	17	23	1/23 (4.3%)	19	4/19 (21.1%)
Hemophilia B	6	9	0/9 (0%)	5	0/5 (0%)
VWD	23	36	5/36 (13.9%)	31	4/31 (12.9%)
VWD type 1	17	28	4/28 (14.3%)	25	4/25 (16.0%)
VWD type 2 (non-B subtypes)	4	6	1/6 (16.7%)	4	0/4 (0%)
VWD type 2B	2	2	0/2 (%)	2	0/2 (0%)
Combined hemophilia A + VWD	3	4	0/4 (0%)	4	1/4 (25.0%)
Factor VII deficiency	3	4	0/4 (0%)	4	0/4 (0%)
Factor XI deficiency	1	2	0/2 (0%)	2	1/2 (50.0%)
Factor XIII deficiency	2	3	0/3 (0%)	3	2/3 (66.7%)
Platelet type: VWD	1	1	0/1 (0%)	1	0/1 (0%)

PPH, postpartum hemorrhage; VWD, von Willebrand disease.

**Table 3:** OR and CI for primary and secondary PPH by mode of delivery.

Mode of delivery	Primary PPH (*n* = 6) OR (95% CI)		Secondary PPH (*n* = 12) OR (95% CI)	
Vaginal deliveries vs cesarian deliveries	VD (*n* = 4/60)	0.7 (0.1-3.4)	VD (*n* = 11/54)	2.6 (0.4-16.4)
	CD (*n* = 2/22)		CD (*n* = 1/15)	
Unplanned vs planned deliveries	Unplanned (*n* = 2/44)	0.5 (0.1-2.3)	Unplanned (*n* = 7/39)	1.1 (0.3-3.7)
	Planned (*n* = 4/38)		Planned (*n* = 5/30)	

CD, cesarian delivery; OR, odds ratio; PPH, postpartum hemorrhage; VD, vaginal delivery.

**Table 4:** Characteristics of individuals who developed PPH by bleeding disorder, clotting factor levels, mode of delivery, and peripartum hemostatic therapy.

Individuals^a^	Bleeding disorder and clotting factor levels (IU/mL)	Mode of delivery	Peripartum hemostatic therapy	PPH
1	Hemophilia A Baseline: FVIII 0.61 At T3: FVIII: 1.26	SVD	None	Secondary
2a	Hemophilia A Baseline: FVIII 0.58 At T3: FVIII 1.47	SVD	None	Primary + secondary
2b	Hemophilia A Baseline: FVIII 0.58 At T3: FVIII 1.33	IOL for LGA	None	Secondary
2c	Hemophilia A Baseline: FVIII 0.58 At T3: ND	IOL for LGA	None	Secondary
3	FXI deficiency Baseline: FXI 0.45 At T3: FXI 0.57	Emergent CD for fetal heart rate	None	Secondary
4	VWD type 1 Baseline: VWF activity 0.11; VWF Ag 0.18; FVIII 0.39 At T3: VWF activity 0.23; VWF Ag 0.23; FVIII 0.44	SVD	Desmopressin 19 μg s.c. once and TXA 1 g i.v. once at onset of labor, then VWF/FVIII concentrates 40-50 units/kg i.v. bolus every 12 h for 2 doses and TXA 1500 mg PO every 8 h for 7 d	Secondary
5	VWD type 1 Baseline: VWF activity 0.44; VWF Ag 0.44; FVIII 0.7 At T3: VWF activity 0.95; VWF Ag 1.01; FVIII 1.26	IOL for hypertension in pregnancy	TXA 1500 mg PO every 8 h for 5 d	Primary
6	VWD type 2A Baseline: VWF activity < 0.1; VWF Ag 0.41; FVIII 0.48 At T3: VWF activity < 0.1; VWF Ag 0.47; FVIII 0.23	Emergent CD for failure to progress and placenta previa	VWF/FVIII concentrate continuous infusion at 3 units/kg/h for 24 h, then 40 units/kg boluses every 12 h for 4 doses TXA 1500 mg PO every 8 h for 10 d	Primary (placenta previa)
7	VWD type 2 non-B subtype and Hemophilia A Baseline: VWF activity 0.28; VWF Ag 0.6; FVIII ND At T3: VWF activity 0.17; VWF Ag 0.89; FVIII 0.85	SVD	VWF/FVIII concentrate 60 units/kg i.v. bolus at onset of labor and then 40 units/kg every 12 h for 3 doses + TXA 1500 mg PO every 8 h for 7-14 d	Secondary
8	VWD-1 Baseline: VWF activity 0.26; VWF Ag 0.17; FVIII 0.31 At T3: VWF activity 1.06; VWF Ag 0.92; FVIII 0.99	IOL for PROM	VWF/FVIII concentrate 25 units/kg for 1 dose + TXA 1000 mg PO every 8 h for 3 doses	Primary + secondary
9a	FXIII deficiency Baseline: ND At T3: ND	IOL for LGA	None	Secondary
9b	FXIII deficiency Baseline: ND At T3: FXIII 0.42	SVD	None	Secondary
10	VWD type 1 Baseline: VWF activity 0.34; VWF Ag 0.37; FVIII 0.49 At T3: ND	IOL for PROM	None	Primary + secondary (retained placenta)
11	VWD type 1 Baseline: VWF activity 0.46; VWF Ag 0.53; FVIII 0.54 At T3: VWF activity 1.34; VWF Ag 1.20; FVIII 1.55	SVD	Desmopressin 20 μg i.v. 1-time dose at onset of labor + TXA 1.5 g PO every 8 h for 5 d	Secondary
12	VWD type 1 Baseline: ND At T3: VWF activity 0.82; VWF antigen 1.19; FVIII 2.20	Planned CD	TXA 1000 mg i.v. given 60-min prior to CD and then 1500 mg PO every 8 h for 14 d	Primary

Ag, antigen; CD, cesarian delivery; F, factor; IOL, induction of labor; IV, intravenous; LGA, large for gestational age; ND, no data; PO, per oral; PPH, postpartum hemorrhage; PROM, premature rupture of membranes; SC, subcutaneous; SVD, spontaneous vaginal delivery; T3, third trimester; TXA, tranexamic acid; VWD, von Willebrand disease; VWF, von Willebrand factor.

a Each number represents a person with a bleeding disorder who developed PPH during their delivery. When PPH complicated multiple deliveries, each delivery was represented by a lowercase letter.

In this cohort, 40 subjects did not receive peripartum hemostatic therapies. Of these, 5% (2/40) developed primary PPH, and 21.9% (7/32) developed secondary PPH. Hemostatic therapies, consisting of factor concentrates or desmopressin with tranexamic acid (TXA), were initiated in 27 deliveries. In these cases, primary and secondary PPH were reported in 7.4% (2/27) and 16.7% (4/24), respectively. Hemostatic therapies were initiated at the onset of labor before CD or neuraxial anesthesia with variable dosing and frequency of administration. Postpartum duration of TXA was heterogeneous, ranging from 24 hours to 14 days. For 11 subjects who received TXA alone, primary and secondary PPH were reported in 18.1% (2/11) and 10% (1/10), respectively. Four subjects received factor concentrates alone without developing primary or secondary PPH, although data for secondary PPH were missing for 1 patient. PPH outcomes in relation to hemostatic therapies are detailed in Supplementary Material 4.

Reassuringly, we did not observe maternal or neonatal deaths. One patient with VWD type 2A received 2 units of PRBCs (P6, Table 4). No subjects required platelet transfusions. Maternal bleeding complications other than PPH included a vaginal hematoma in a woman with VWD type 1 and a wound hematoma at the site of sutured vaginal tear in a woman with hemophilia A, both following SVD with factor levels above 0.50 IU/mL. For neonatal outcomes, only 1 neonate had a suspected intracranial hemorrhage based on head imaging. He had severe hemophilia A and was born by vaginal delivery following IOL. He received recombinant FVIII concentrates promptly and was transferred in stable condition to the pediatric hospital without subsequent adverse outcomes.

PPH rates were numerically lower with CDs and highest with IOL, but no statistically significant differences were found compared to vaginal deliveries. Recent observational studies in the general population have also shown lower rates of PPH with CDs [5,6,13]. However, Wolf et al. [14] observed a higher frequency of primary PPH for persons with bleeding disorders who had an elective or emergent CD, but secondary PPH was only noted with vaginal deliveries. An Australian observational study of 23 deliveries in subjects with VWD found primary PPH in 44% of their cohort and more frequently with CD, but the differences were not statistically significant [15]. Both studies used the World Health Organization definition of primary PPH (blood loss >500 mL in the first 24 hours) and secondary PPH (abnormal bleeding between 24 hours and up to 6 weeks postpartum), which may have contributed to the differences in findings. Planned CD for this patient population may have an advantage due to its predictability in scheduling the procedure and allowing for appropriate care coordination and availability of laboratory, medications, and expert care, which fits with international surgical standards for persons with hemophilia [16]. However, SVD and IOL offer less invasive options with avoidance of surgical risks and abnormal placentation risks associated with repeated CD. While rates of primary PPH in this cohort may be similar to those in persons without bleeding disorders [5,17], minimizing the risk of secondary PPH in persons with bleeding disorders requires attention to factors beyond the mode of delivery, specifically the need for clotting factor replacement therapy after discharge.

In this study, secondary PPH was primarily noted in subjects with hemophilia A or VWD type 1. A matched controlled study from the United Kingdom showed that persons with bleeding disorders had a higher frequency of PPH compared to those without bleeding disorders, with subjects with hemophilia A having higher odds of primary PPH despite having levels over 0.50 IU/mL at the time of delivery and not receiving hemostatic prophylaxis [14]. A retrospective study from the United States Nationwide Inpatient Sample database comparing maternal outcomes in subjects with and without VWD reported a 6% incidence of PPH in subjects with VWD with an OR of 1.5 (95% CI, 1.1-2.0) [1]. Although subjects with VWD were more likely to require transfusions, the study did not specify whether it was PRBCs or factor concentrates [18]. FVIII and VWF activity levels below 0.50 IU/mL are accepted target thresholds for administering prophylactic hemostatic therapy when managing subjects with VWD and hemophilia A [[19], [20], [21]]. Normalization of FVIII levels was previously thought to abrogate the risk of PPH [22]; however, subsequent studies have shown that there remains a clear risk of primary and secondary PPH even with normalization of FVIII levels in the third trimester [20,[23], [24], [25], [26]]. Therefore, higher target of factor levels may be needed in the peripartum and postpartum periods as factor levels also rise physiologically in persons without bleeding disorders. However, the optimal target levels remain elusive, and current approaches may not be optimal. Further studies are needed to guide practice changes in peripartum prophylaxis in subjects with VWD and hemophilia A.

Observing fewer secondary PPH in subjects who received hemostatic therapies is a notable finding; however, the heterogeneity of bleeding disorders and treatment regimens limits its generalizability. Given the high rates of secondary PPH observed in our cohort, it may be imperative to consider the use of antifibrinolytic agents postpartum, regardless of factor levels. TXA is a safe, well-tolerated, cost-effective [27], and clinically effective option for preventing PPH without risks of thrombosis [28,29].

Our study has limitations. The statistical power was limited due to a small sample size in a single center and the use of estimated blood loss and clinical documentation to assess PPH. The lack of complete data on secondary PPH also limits its accuracy. Additionally, the lack of available information on race and ethnicity impedes identifying potential health disparities. Furthermore, the absence of standardized protocols for deliveries and hemostatic therapies may influence the results. Thus, there is a need for multicenter studies and registries to provide more data and guide shared decision-making for pregnant persons with bleeding disorders, similar to efforts seen for persons with hemophilia. The definitions of secondary PPH were not standardized, which can lead to over- or underestimation. Further efforts are needed to standardize definitions of secondary PPH for clinical and research use.

In summary, we found high rates of primary and secondary PPH in pregnant persons with bleeding disorders, which was not influenced significantly by delivery modes. The rate of secondary PPH was higher than anticipated at 17.4%, which is concerning as it is often diagnosed after discharge. High alert is needed to avoid delaying treatments, and extended antifibrinolytic therapy may be considered. Primary PPH still occurred in subjects with hemophilia A and VWD despite factor levels exceeding the recommended threshold of 0.50 IU/mL, which raised questions about optimal factor level targets. Larger multicenter studies are needed to confirm these findings.
