# Supplementary material for: Bleeding disorders and postpartum hemorrhage by mode of delivery: a retrospective cohort study
Source: Res Pract Thromb Haemost. 2023 Apr 26;7(4):100166. doi: 10.1016/j.rpth.2023.100166 (PMC10225912; doi:10.1016/j.rpth.2023.100166)
Supplement: Supplementary Tables [file mmc1.docx]

**Appendix 1. Inclusion and Exclusion Criteria for Study Subjects**

Subjects were included in our study if they met both of the following criteria:

1. They had a known and specific diagnosis of bleeding disorder, which included
   1. Subjects with hemophilia A (factor VIII deficiency) or hemophilia B (factor IX deficiency) if they were obligate carriers or symptomatic, regardless of the documented baseline factor level
   2. Subjects with von Willebrand disease (VWD) if their von Willebrand factor (VWF) activity was less than 0.30 IU/mL, genetic testing consistent with VWD, or if they had a documented positive bleeding history with VWF activity levels between 0.30-0.50 IU/mL based on laboratory testing prior or after their pregnancy
   3. Subjects with rare bleeding disorders such as fibrinogen disorders, deficiencies in coagulation factors II, V, VII, X, XI, and XIII, inherited platelet function defects, and disorders of hyperfibrinolysis
2. Delivered at TOH between January 1, 2010 and July 15, 2021. This period was selected based on data availability in our electronic medical record.

Persons with bleeding disorders not otherwise specified or with bleeding of an unknown cause were excluded.

**Appendix 2. Frequency of primary and secondary postpartum hemorrhage (PPH) by mode of delivery.**

|  | **Primary PPH** | **Secondary PPH** |
| --- | --- | --- |
| Overall | 6/82 (7.3%) | 12/69 (17.4%) |
| Spontaneous vaginal delivery | 1/36 (2.8%) | 6/32 (18.8%) |
| Induction of labor with intended vaginal delivery | 3/24 (12.5%) | 5/22 (22.7%) |
| Emergent Cesarian Delivery (CD) | 1/8 (12.5%) | 1/7 (14.3%) |
| Planned CD | 1/14 (7.1%) | 0/8 (0%) |

**Appendix 3. Characteristics of the deliveries who did not have secondary PPH data**

| **Characteristics** | **No. (%)**  **N =13 deliveries** |
| --- | --- |
| **Maternal age at time of delivery (years)** |  |
| 20 – 24 | 1 (7.7) |
| 25 – 29 | 2 (15.3) |
| 30 – 34 | 6 (46.2) |
| > 35 | 4 (30.8) |
| **Gestational age at time of delivery (weeks)** |  |
| 37+0 – 39+9 | 9 (69.2) |
| ≥ 40 | 4 (30.8) |
| **Mode of delivery** |  |
| Spontaneous vaginal delivery | 4 (30.8) |
| Induction of labor with intended vaginal delivery | 2 (15.3) |
| Planned cesarian delivery | 6 (46.2) |
| Emergent cesarian delivery | 1 (7.7) |
| **Bleeding Disorders** |  |
| Hemophilia | 8 (61.5) |
| - Hemophilia A | 4 (30.8) |
| - Hemophilia B | 4 (30.8) |
| Von Willebrand disease (VWD) | 5 (38.5) |
| - VWD type 1 | 3 (23.1) |
| - VWD type 2 (non-B subtypes) | 2 (15.3) |
| **Primary PPH** | 0 (0) |

**Appendix 4. Frequency of primary and secondary postpartum hemorrhage (PPH) in relation to peripartum hemostatic management.**

|  | **Primary PPH**  **N (%)** | **Secondary PPH**  **N (%)** | **Additional descriptors on therapies and factor activity assays with N representing each pregnancy as separate event** |
| --- | --- | --- | --- |
| Factor concentrates* alone | 0/4 (0%) | 0/3 (0%) | Hemophilia B (N=1):   - SHL rFIX CFC for FIX:C < 0.50 IU/mL   VWD type 1 (N=2):   - Desmopressin for VWF activity between 0.50-0.99 IU/mL and FVIII:C > 1.00 IU/mL   VWD type 2N (N=1):   - FVIII/VWF CFC for VWF activity > 1.00 IU/mL and FVIII:C < 0.50 IU/mL |
| Tranexamic acid (TXA) alone | 2/11 (18.1%) | 1/10 (10%) | Hemophilia A (N=4):   - FVIII:C between 0.50-0.99 IU/mL (N=1) - FVIII:C > 1.00 IU/mL (N=2) - No FVIII:C measured in pregnancy (N=1)   VWD type 1 (N=5):   - VWF activity < 0.50 IU/mL and FVIII:C between 0.50-0.99 IU/mL (N=1) - VWF activity between 0.50-0.99 IU/mL and FVIII > 1.00 IU/mL (N=3) - VWF activity and FVIII:C > 1.00 IU/mL (N=1)   VWD type 2b (N=1)   - VWF activity and FVIII:C > 1.00 IU/mL; Platelet count estimated manually at 40 x 10^9^/L due to clumping   Platelet-type VWD (N=1)   - VWF activity and FVIII:C > 1.00 IU/mL; Platelet count at 119 x 10^9^/L |
| Factor concentrates* and TXA | 2/27 (7.4%) | 4/24 (16.7%) | Hemophilia A carrier and VWD (N=4):   - FVIII/VWF CFC for VWF activity < 0.50 IU/mL and FVIII:C between 0.50-0.99 IU/mL (N=2) - Desmopressin for VWF activity and FVIII:C < 0.50 IU/mL (N=1) - Desmopressin for VWF activity and FVIII:C between 0.50-0.99 IU/mL   Hemophilia B (N=4)   - SHL rFIX CFC for FIX:C < 0.50 IU/mL (N=3) - SHL rFIX CFC for FIX:C between 0.50-0.99 IU/mL (N=1)   VWD type 1 (N= 10)   - FVIII/VWF CFC for VWF activity and FVIII:C < 0.50 IU/mL (N=1) - FVIII/VWF CFC for VWF activity < 0.50 IU/mL and FVIII:C between 0.50-0.99 IU/mL (N=5) - FVIII/VWF CFC for VWF activity > 1.00 IU/mL and FVIII:C between 0.50-0.99 IU/mL (N=1) - Desmopressin for VWF activity and FVIII:C > 1.00 IU/mL (N=3)   VWD type 2 non-B (N=5)   - FVIII/VWF CFC for VWF activity and FVIII:C < 0.50 IU/mL (N=1) - FVIII/VWF CFC for for VWF activity < 0.50 IU/mL and FVIII between 0.50-0.99 IU/mL (N=4)   VWD type 2B (N=1)   - FVIII/VWF CFC for VWF activity < 0.50 IU/mL and FVIII > 1.00 IU/mL   Factor VII deficiency (N=2)   - PCC 40 mL prior to epidural for FVII:C at 40 IU/mL   Factor XIII deficiency (N=1)   - FXIII CFC as per subject’s usual prophylaxis regimen with FXIII:C > 1.00 IU/mL at delivery |
| No hemostatic therapies | 2/40 (5.0%) | 7/32 (21.9%) | Hemophilia A (N=19)   - FVIII:C between 0.50-0.99 IU/mL (N=6) - FVIII:C > 1.00 IU/mL (N=11) - No FVIII:C measured in pregnancy (N=2)   Hemophilia B (N=4)   - FIX:C between 0.50-0.99 IU/mL (N=4)   VWD type 1 (N=11)   - VWF activity < 0.50 IU/mL and FVIII > 1.00 IU/mL (N=1) - VWF activity between 0.50-0.99 IU/mL and FVIII > 1.00 IU/mL (N=4) - VWF activity and FVIII:C between 0.50-0.99 IU/mL (N=1) - VWF activity and FVIII:C > 1.00 IU/mL (N=3) - No VWF activity or FVIII:C measured (N=2)   Factor VII deficiency (N=2) with FVII:C between 0.50-0.99 IU/mL  Factor XI deficiency (N=2) with FIX:C < 0.50 IU/mL  Factor XIII deficiency (N=2) with FXIII < 0.50 IU/mL (N=1) and none measured (N=1) |

* Factor concentrates included administration of either desmopressin or clotting factor concentrates.

Abbreviations: FIX:C: Factor IX activity assay; FVII:C: Factor VII activity assay; FVIII:C: Factor VIII activity assay; FXIII:C: Factor XIII activity assay; PCC: Prothrombin complex concentrate; SHL: Standard half-life; rFIX: recombinant factor IX; CFC: Clotting factor concentrates; VWD: von Willebrand disease; VWF: von Willebrand Factor
